# Hepatitis C Virus Screening of High-Risk Patients in a Canadian Emergency Department

**DOI:** 10.1155/2020/5258289

**Published:** 2020-02-18

**Authors:** Kelsey Ragan, Anjali Pandya, Tristan Holotnak, Katrina Koger, Neil Collins, Mark G. Swain

**Affiliations:** ^1^Department of Emergency Medicine, University of Calgary, Calgary, AB, Canada; ^2^Cumming School of Medicine, University of Calgary, Calgary, AB, Canada; ^3^Division of Gastroenterology, Department of Medicine, University of Calgary, Calgary, AB, Canada

## Abstract

**Background:**

Approximately 0.7% of the Canadian population is infected with hepatitis C virus (HCV), and many individuals are unaware of their infection. Our objectives were to utilize an emergency department (ED) based point-of-care (POC) HCV screening test to describe our local population and estimate the proportion of high-risk patients in our population with undiagnosed HCV.

**Methods:**

A convenience sample of medically stable patients (≥18 years) presenting to a community ED in Calgary, AB, between April and July 2018 underwent rapid clinical screening for HCV risk factors, including history of injection drug use, healthcare in endemic countries, and other recognized criteria. High-risk patients were offered POC HCV testing. Antibody-positive patients underwent HCV-RNA testing and were linked to hepatology care. The primary outcome was the proportion of new HCV diagnoses in the high-risk population.

**Results:**

Of the 999 patients screened by survey, 247 patients (24.7%) were high-risk and eligible for testing. Of these, 123 (49.8%) were from HCV-endemic countries, while 63 (25.5%) and 31 (12.6%) patients endorsed a history of incarceration and intravenous drug use (IVDU), respectively. A total of 144 (58.3%) eligible patients agreed to testing. Of these, 6 patients were POC-positive (4.2%, CI 0.9–7.4%); all 6 had antibodies detected on confirmatory lab testing and 4 had detectable HCV-RNA viral loads in follow-up. Notably, 103 (41.7%) patients declined POC testing. *Interpretation.* Among 144 high-risk patients who agreed to testing, the rate of undiagnosed HCV infection was 4.2%, and the rate of undiagnosed HCV infection with detectable viral load was 2.8%. Many patients with high-risk clinical criteria refused POC testing. It is unknown if tested and untested groups have the same disease prevalence. This study shows that ED HCV screening is feasible and that a small number of previously undiagnosed patients can be identified and linked to potentially life-changing care.

## 1. Introduction

Hepatitis C virus (HCV) infection represents a significant public health problem in Canada. Over 220,000 Canadians are living with chronic hepatitis C infection, with approximately 44% of individuals undiagnosed or unaware of their disease status [1–3]. Current guidelines suggest screening individuals at high risk, including those from endemic countries, injection drug users, and individuals with a history of incarceration [4,5]. Studies from US urban emergency departments (ED) estimate rates of undiagnosed HCV infection from 7.3 to 26%, making the ED a potentially efficacious setting for screening [6–10]. New, highly effective treatment regimens for HCV have improved treatment tolerance and are associated with high rates of virologic cure. In Canada, healthcare costs associated with chronic HCV infection are projected to rise by 60% until 2032, making the cost savings of an effective cure high-yield [11]. The potential for long-term reduction in cirrhosis complications, including hepatocellular carcinoma, makes it important to identify settings for screening and testing of individuals who may benefit from HCV treatment [12–14].

Several studies have estimated HCV seroprevalence in ED populations; however, most are reflective of US urban or inner-city populations where social determinants of health, including access to health care, healthcare delivery models, and risk factor distribution differ from Canada [15–20]. We identified Canadian studies that estimated prevalence amongst inpatients, but we found no published descriptions of Canadian ED HCV screening protocols or seroprevalence estimates [21]. We chose to study an ED-based HCV screening process because the ED is often the primary source of healthcare for many high-risk and marginalized patients. By providing immediate results, we felt point-of-care (POC) testing would reduce loss to follow-up and enhance uptake in a rapid turnover ED environment.

The objective of this study was to implement a POC testing strategy, to describe our local patient population and to explore the feasibility of this screening strategy during an ED visit. In addition, we sought to estimate the prevalence of undiagnosed chronic HCV infection in a convenience sample of high-risk patients presenting to our urban emergency department.

## 2. Methods

### 2.1. Study Design and Setting

This was an observational pilot study of a convenience sample of patients at a community hospital ED in Calgary, Alberta, Canada, over a 3-month period between April and July 2018. The annual ED census is approximately 82,000 patients, and the department is located in an area that serves racially and socioeconomically diverse populations, including a large new-immigrant population. The study protocol was approved by the University of Calgary Conjoint Health Research Ethics Board. Operational approval was provided by Alberta Health Services.

### 2.2. Participants

During study hours (1000–2200), research assistants (RAs) approached medically stable adult patients (age ≥ 18 years) for enrollment. Study hours coincided with the periods of highest ED patient arrival. RAs used the electronic patient tracking board to track patient location and to determine Canadian Triage Acuity Scale (CTAS) score. Patients with a psychiatric chief complaint, a CTAS score of 1 or 2, or patients not yet assigned to a treatment space were not approached for enrollment. Patients were also excluded from possible enrollment if they were known to lack capacity and did not have an alternate decision maker present. Our protocol allowed treating physicians or bedside nurses to exercise discretion and exclude patients if they had clinical concerns or if study participation would potentially interfere with clinical care.

### 2.3. Screening and Identification of High-Risk Patients

Patients were enrolled using a two-step consent process. First, RAs approached patients, introduced the study purpose, and obtained verbal consent for a brief self-administered questionnaire (see [Supplementary-material supplementary-material-1] in Supplementary Materials). This questionnaire served as both a screening tool and a data collection form. It was drafted in English to reflect previously validated high-risk criteria for HCV as described by current Canadian and US guidelines and was professionally translated into the three most commonly spoken languages within the catchment area (Arabic, Punjabi, and Simplified Chinese) based upon an ED census administrative review [4,5]. RAs also had access to a professional telephone translation service (provided by Language Line Services) if needed. Consenting patients returned completed surveys to the RA.

### 2.4. Testing of High-Risk Patients

**Figure 1:**
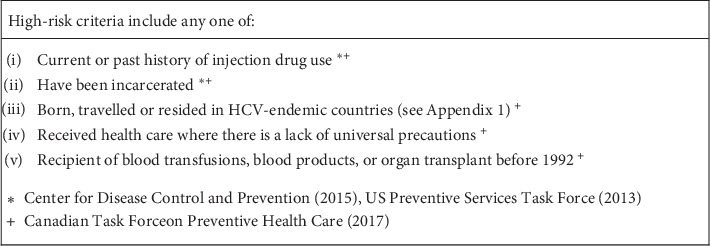
High-risk criteria for patient screening.

Patients who self-reported one or more high-risk criteria (Figure 1), and who did not self-report a positive HCV test within the past year, were offered POC HCV testing. In the case of a self-reported positive result within the past year, no data was collected with respect to treatment and these patients were not retested as it was presumed they were linked to care by their testing provider.

After obtaining written informed consent, RAs completed testing with a finger-prick blood sample using Oraquick® HCV Rapid Antibody Test. All RAs were trained in person on how to perform testing. Oraquick® HCV Rapid Antibody Test has a reported sensitivity and specificity of 100% (95%CI 97–100%, 99.2–100%, respectively) (Orasure Technologies Inc.) [22].

Positive rapid antibody POC results were interpreted by the RA but disclosed to the patient by the treating emergency physician with the aid of a standardized information sheet. Treating physicians also provided patients with HCV education including written materials [23]. All patients with a positive POC test had blood samples for HCV-RNA load and genotype drawn by their bedside nurse during their ED stay based on laboratory requisitions prefilled by one of the study investigators (AP).

All POC-positive patients were referred to the University of Calgary Viral Hepatitis Clinic to complete linkage to care and for consideration of antiviral treatment. Antibody-positive patients without an established primary care provider were also referred to the local primary care network. No patient was discharged or left the ED prior to the result of the POC test being available, and admitted patients underwent the same referral process.

### 2.5. Data Collection and Processing

Data collection was completed by trained emergency department research assistants (RAs). Each RA received standardized training by the primary investigators on the consent process, data collection, and POC testing in accordance with REB requirements and operational approval. In addition, RAs had prior experience recruiting patients in ED-related studies and were drawn from a pool of RAs within a preexisting ED research program.

All data points were recorded by the RAs into a secure database (Excel). Data on risk factors, as well as access to primary and alternate care, were collected on the self-administered survey. This data was not recorded on the patient chart. HCV antibody test results were entered directly into the study database by RAs, while HCV-RNA and genotyping test results were accessed later from the electronic medical record by one of the study clinicians (AP). Follow-up attendance at the first scheduled appointment in the Viral Hepatitis Clinic was confirmed by clinic staff. Hard copy documentation was reviewed in the case of missing data only.

### 2.6. Outcome Measures

Our primary outcome was the number and proportion of high-risk patients with self-report of previously unknown or negative disease status, who tested positive for HCV by rapid point-of-care antibody test. Secondary outcomes included the proportion of eligible, screened patients who met high-risk criteria for HCV infection and the proportion of POC-positive patients who had detectable viral load (as determined by HCV-RNA testing). In addition, we determined the proportion of high-risk patients with a regular primary care provider, as well as the number of patients who had accessed alternate care settings (e.g., walk-in clinics and urgent care) within the previous year. Finally, we evaluated the number of newly identified HCV-positive patients who attended follow-up care for HCV management.

### 2.7. Data Analysis

Descriptive analyses were performed for all variables using patient level data. Categorical data are reported as proportions with 95% confidence intervals and continuous data are reported as means with standard deviations. The effect of defined risk factors was assessed using risk ratios. We did not perform an a priori sample size calculation as this was intended as a descriptive analysis. All statistical analyses were performed using Excel (Version 16.17, Microsoft).

## 3. Results

**Figure 2:**
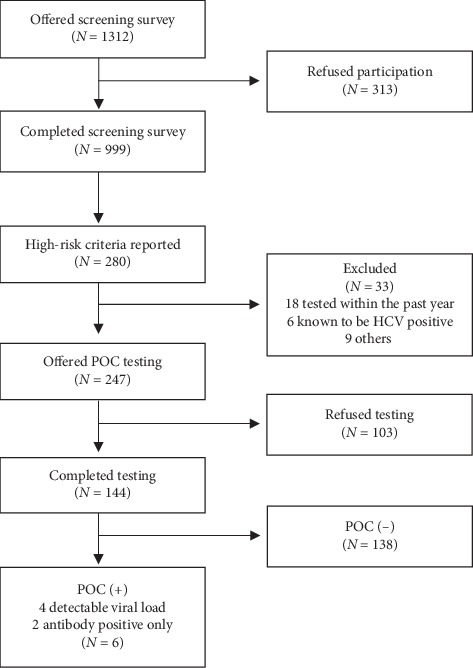
HCV risk factor screening and POC testing flowchart.

As illustrated in Figure 2, 1312 patients were approached to complete the screening questionnaire, 999 (76.1%) completed it, and 280 respondents (28.0%, 95% CI 25.2%–30.8%) had at least one high-risk criterion. In the latter high-risk group, 18 patients (6.4%) had tested positive for HCV in the previous year, 6 (2.1%) were known to be HCV-positive, and 9 (3.2%) were excluded for other reasons. Only 144 of the 247 eligible high-risk patients (58%) consented to POCT and six of these (4.2%; 95% CI 0.9%–7.4%) tested positive for HCV antibodies. Of the 6 HCV antibody-positive patients, four tested positive for HCV-RNA indicating active viremia and the remaining two were confirmed to be serologically antibody-positive on provincial lab testing, indicating a previously cleared infection rather than a false-positive POCT. All represented new diagnoses, and all were referred for follow-up as per the study protocol.

Table 1 shows the demographics and characteristics of our cohort. Of the 999 patients screened, 464 were male and 535 were female (46.4 and 53.6%). The mean age was 53.8 years.

Of 247 high-risk patients eligible for HCV testing, 123 (49.8%) came from HCV-endemic countries (see [Supplementary-material supplementary-material-1] in Supplementary Materials), most commonly the Philippines (46/123, 37.4%), Pakistan (21/123, 17.1%), and China (10/123, 8.1%). Six of 247 high-risk patients (2.4%) admitted to current, and 25 (10.1%) to past, injection drug use. A total of 73 patients (25.5%) were previously incarcerated and 41 (16.5%) had received blood transfusions. Two patients endorsed a history of one or more high-risk factors (blood transfusion, incarceration, or IVDU), but did not disclose which risk factor(s) they had been exposed to. Both patients consented to testing and both tested negative.

In the group of 144 high-risk patients who consented to testing, 6 (4.2%) had positive POC tests. Rates of HCV test positivity were 1.6% (1/63) in patients with a geographic exposure, 15.4% (4/26) with any injection drug use history, 7.3% (3/41) with an incarceration history, and 3.7% (1/28) with a transfusion history. Patients who refused testing were significantly more likely to have been from an endemic country and less likely to have endorsed a history of IVDU.  Table 2 summarizes unadjusted risk ratios for each of the high-risk criteria, excluding the two patients who did not disclose specifics.

Most of the patients who were screened reported accessing a primary care provider (83.4%), a walk-in clinic (36.2%), an ED (55.1%), or an urgent care center (13%) within the prior year. Only 45 patients (0.5%) had not seen a healthcare provider within the previous year. Two patients did not answer the questions relating to alternate care providers. At the time of writing, 5 of the 6 patients who tested antibody-positive had attended their hepatology follow-up appointment, including 3 of the 4 patients with newly diagnosed HCV infection.

## 4. Discussion

We successfully implemented a risk factor based point-of-care (POC) screening strategy designed to identify patients with previously unrecognized hepatitis C virus (HCV) infection presenting to a Canadian emergency department. In our study population, 28% of patients screened had high-risk HCV predictors, and six of the 144 high-risk patients tested positive for HCV antibodies (4.2%). All of the positive POC results were true-positive results, with antibodies confirmed on subsequent serologic testing. This represented 4 newly diagnosed cases of HCV infection (2.8%) and 2 patients with cleared past infections. A significant number of patients approached declined participation in both the screening survey and POC testing. Despite this, a small but clinically important number of HCV-positive patients were identified and linked to care.

The strongest risk factor associated with a new HCV diagnosis in our study was a history of IVDU (RR 10.4, *P*=0.01). This is consistent with prior studies, which have shown IVDU to be the most important risk factor for HCV in countries with low overall prevalence and implemented screening of blood products (such as Canada). However, as the group that refused testing in our population had significantly lower rates of IVDU and higher rates of geographic exposure, the magnitude of this relative risk may represent an overestimate. In addition, as we only sought to test those who had not been previously tested, this may reflect that patients with risk factors such as IVDU have access to testing in alternate settings such as safe injection sites and clinics serving this population.

Relative to prior studies, mainly from urban American centres, we found a lower proportion of unrecognized HCV infection. Our exclusion of the 1945–1965 baby boomer birth cohort as an independent risk factor as well as different patterns of intravenous drug use in Canada compared to the US may have both contributed to the lower than expected rate of previously undiagnosed patients with HCV [24]. Other studies using a similar targeted, opt-in approach have reported higher HCV positivity rates between 7.3% and 11.1%, which may be due to their inclusion of the baby boomer cohort as a risk factor [6,8,9]. More recently an ED-based, nontargeted, opt-out study found an HCV-positive rate of 7.7%, revealing a significant number of infected patients who did not have traditional risk factors [25]. Additionally, our findings may be an underestimate given the high rate of refusal, and especially the significantly higher number of immigrant patients amongst those who refused testing, since rates of HCV infection amongst immigrants from endemic countries is estimated to be up to 1.7 times higher than non-immigrants [26].

Importantly, contrary to published US data reporting follow-up rates from 2 to 30% [6–10], we have shown that linkage to care from the ED in the Canadian context is likely to be successful. In comparison, we were able to successfully link 5 of the 6 identified HCV antibody-positive patients.

### 4.1. Limitations

Our study was limited by the use of a convenience sample and a high refusal rate. In combination with our reliance on self-reporting and voluntary participation, this puts our findings at risk of sampling and reporting bias. In addition, we did not have access to patients' medical records to verify reports of prior testing.

Language barriers may have played a role in limiting participation despite the use of translated materials and phone translation, and while we used RAs instead of members of the treating healthcare team to mitigate any perception on the part of patients that their disclosures may impact their care, it is possible that patients remained reticent to disclose risk behavior due to stigmatization. This is a well described barrier to HCV screening, especially amongst immigrant populations, and may explain the higher proportion of immigrants amongst those who refused testing compared to those who accepted testing [27].

Notably, the rate of refusal for both survey participation and point-of-care testing was higher than anticipated. Unfortunately, we do not have detailed data on the reason for refusal for the majority of patients, limiting our ability to conclude whether refusal was related primarily to nonmodifiable patient specific factors or modifiable situational factors. This may suggest that an opt-out strategy, as utilized in other ED screening efforts, as opposed to the opt-in strategy used in this study, would result in higher uptake and therefore a higher rate of identification of at-risk patients [8]. Previous studies of the acceptability of POCT of ED patient populations have demonstrated that the most common reasons for refusal are that patients do not self-assess as being at risk, are apprehensive about blood draws, would prefer to focus on their presenting complaints, and have privacy concerns [6].

### 4.2. Future Directions

Public health initiatives not fitting within the traditional scope of emergency services, such as screening programs for domestic violence, substance use, and communicable disease, have been widely instituted in emergency departments, setting a precedent for HCV screening [28].

Implementing a long-term ED-based screening strategy for HCV, however, would require using existing resources in place of research assistants. Adding to the workflow of frontline staff is a major barrier to implementing screening programs, but it is plausible that routine HCV screening requiring only verbal consent would increase uptake compared to the multistep process involved in our research protocol. Beyond this, the responsibility for test follow-up, time constraints, and unfamiliarity with counselling continues to deter many emergency providers from performing tests that are not indicated for acute care provision. Defining a follow-up pathway and providing written patient resources, as we did in our study, may overcome this challenge and should be implemented in any similar screening protocol.

## 5. Conclusions

Our study shows that it is possible to identify patients at high risk of HCV infection as well as to offer testing and facilitate their linkage to specialist care within an unrelated emergency room visit. The high rate of refusal encountered in this study was a large limitation, but we identified and successfully linked to care three new cases of HCV in a single community Canadian ED despite this limitation, suggesting that the true yield of a more effective and widespread ED screening program may be higher. The successful development of an integrated screening program requires further research to maximize uptake and determine the optimal setting, organizational structure, and target population(s).

## Figures and Tables

**Table 1:** Participant demographics.

	Screened	High-risk (tested)	High-risk (refused)	95% CI	*P* value
*N*	999	144	103		
Age: mean (SD)	53.8 (19.6)	53.5 (18.0)	55.8 (19.1)	−2.4, 7.0	0.34
Male sex: *N* (%)	464 (46.4)	77 (59.7)	52 (50.5)	−3.3, 21.4	0.15
Country					
Canada	607 (60.8)	66 (45.8)	33 (32.0)	1.4, 25.4	**0.03**
Endemic	136 (13.6)	63 (43.8)	60 (58.3)	1.9, 26.5	**0.02**
Nonendemic	256 (25.6)	15 (10.4)	10 (9.7)	−7.6, 8.2	0.86
IVDU					
Ever used	40 (4.0)	26 (18.0)	5 (4.9)	5.0, 20.7	**0.002**
Current use	11 (1.1)	5 (3.5)	1 (0.97)	−2.2, 7	0.20
Past use	29 (2.9)	21 (14.6)	4 (3.9)	3.2, 17.8	**0.006**
Blood transfusion	48 (19.4)	28 (19.4)	13 (12.6)	−2.8, 15.6	0.16
Incarceration	73 (7.3%)	41 (28.5)	22 (21.4)	−4.0, 17.5	0.21

**Table 2:** Effect of defined risk factors on the likelihood of testing positive for HCV antibodies by POCT.

Risk factor	Present (*N*)	HCV+	Risk (%)	Absent (*N*)	HCV+	Risk (%)	Difference (%)	RR	*P*
IDU	26	4	15.4	116	2	1.7	13.7	10.4	0.01
Incarceration	41	3	7.3	101	3	3.0	4.3	2.5	0.24
Geographic	63	1	1.6	81	5	6.2	−4.6	0.3	0.17
Transfusion	28	1	3.6	116	5	4.3	−0.7	0.8	0.66

RR = risk ratio.

## Data Availability

The data used to support the findings of this study are available from the corresponding author upon request.

## Supplementary Materials

Supplementary Materials Appendix 1: Screening questionnaire.
