# Supplementary material for: Hepatitis C Virus Screening of High-Risk Patients in a Canadian Emergency Department
Source: Can J Gastroenterol Hepatol. 2020 Feb 18;2020:5258289. doi: 10.1155/2020/5258289 (PMC7049435; doi:10.1155/2020/5258289)
Supplement: Supplementary Materials — Appendix 1: Screening questionnaire. [file 5258289.f1.pdf]

APPENDIX 1: Screening Questionnaire

HEPATITIS C IN THE EMERGENCY DEPARTMENT

We are doing a study at the <study center> screening for Hepatitis C virus infection in emergency department patients. This short survey is **voluntary** and should take **less than 5 minutes** to complete. All answers are strictly **confidential** between your doctors and the research team.

1. Age: ________years

2. Where were you born (city, country)?
   ____

3. Have you ever received health or dental care outside of Canada?

   - ☐ NO
   - ☐ YES
     - *If YES* – which country? __________________
     - *If YES* – did you receive a blood transfusion or organ donation?
       - ☐ Yes
       - ☐ No

4. Do any of the following statements apply to you:

- ➢ *I have been to jail*
- ➢ *I have used injection drugs*
- ➢ *I have received a blood transfusion or organ transplant in North America before 1992*

  - ☐ Yes, one or more of the above apply
  - ☐ No, none of the above apply

5. Have you ever been tested for Hepatitis C virus?
   - ☐ YES
     - *If Yes* – what were the results?
       - ☐ Positive
       - ☐ Negative
       - ☐ I don't know
   - ☐ NO
   - ☐ I don't know

6. Do you have a family doctor that you see regularly?
   - ☐ YES
   - ☐ NO

7. Please check any locations that you have received care in the past 12 months:

   - ☐ None
   - ☐ Regular family doctor
   - ☐ Walk in clinic doctor
   - ☐ CUPS clinic
   - ☐ Urgent care (Sheldon Chumir or South Calgary Urgent Care)
   - ☐ Emergency Department
   - ☐ Other, please specify: ____

Thank you for your participation

(NOT GIVEN TO PATIENT)

FOR RESARCH ASSISTANT USE ONLY

If the patient has indicated YES to survey question #4 please verbally confirm which risk factor(s) is (are) relevant:

- ☐ Current use of injection drugs
- ☐ Past use of injection drugs
- ☐ History of incarceration
- ☐ Blood transfusion in North America before 1992
- ☐ Organ donation recipient in North America before 1992
- ☐ Patient not willing to disclose
